# Genetic Diversity, Recombination, and Divergence in Animal Associated *Penicillium dipodomyis*

**DOI:** 10.1371/journal.pone.0022883

**Published:** 2011-08-05

**Authors:** Daniel A. Henk, Matthew C. Fisher

**Affiliations:** Department of Infectious Disease Epidemiology, Imperial College, London, United Kingdom; New York State Health Department and University at Albany, United States of America

## Abstract

*Penicillium dipodomyis* is thought to be an exclusively asexual fungus associated with Kangaroo Rats, *Dipodomys* species, and is unique among *Penicillium* species in growing at 37°C but producing no known toxins. Lack of recombination within *P. dipodomyis* would result in limited adaptive flexibility but possibly enhance local adaptation and host selection via maintenance of favourable genotypes. Here, analysis of DNA sequence data from five protein-coding genes shows that recombination occurs within *P. dipodomyis* on a small spatial scale. Furthermore, detection of mating-type alleles supports outcrossing and a sexual cycle in *P. dipodomyis*. *P. dipodomyis* was a weaker competitor in *in vitro* assays with other *Penicillium* species found in association with Kanagaroo rats. Bayesian species level analysis suggests that the *P. dipodomyis* lineage diverged from closely related species also found in cheek pouches of Kangaroo Rats and their stored seeds about 11 million years ago, a similar divergence time as *Dipodomys* from its sister rodent taxa.

## Introduction

The fungus *Penicillium dipodomyis* is found in desert environments, on hoarded seeds and in cheek pouches of North American Kangaroo Rats (*Dipodomys* species). *P. dipodomyis* is closely related to the food production fungus *Penicillium nalgiovense* common in cheese and meat, and to the ubiquitous fungus used in the industrial production of penicillin, *Penicillium chrysogenum*. *P. dipodomyis* has no known sexual stage, and its ecology is poorly known, but several authors have postulated that this species is one of several *Penicillium* specially adapted to live with Kangaroo Rats and their seed caches [1], [2]. *Dipodomys spectabilis* has shown a preference for intermediately mouldy seeds, and they even manage seed stores to the benefit of growing intermediate levels of fungal growth [3], [4].

*Penicillium* species in the subgenus *Penicillium* frequently colonize seeds, but they rarely grow at mammalian body temperatures, with four (*P. chrysogenum*, *P. confertum*, *P. flavigenum*, and *P. dipodomyis*) of the sixty described species actively growing at 37°C [5]. Growth at body temperature may allow these species to gain an advantage in colonizing cheek pouches and gaining early access to stored seeds. *Penicillium* species' ability to compete with other fungi and bacteria for food resources can be enhanced via production of secondary metabolites, but *P. dipodomyis* produces fewer metabolites than its close relatives growing at or near mammalian body temperature. Early access, and abundance could overcome many competitive effects and host mammal effects could play a role in structuring the fungal diversity in the cheek pouches.

Adaptation to hosts is suspected to be a prominent component of the ecological factors that can lead to sympatric speciation when recombination is also associated with host selection [6], and among fungi, host specialization is frequently cited as the major factor enabling sympatric speciation [7]. Fungi have many mating systems that can include varieties of selfing and outcrossing and combinations of the two, but many species have been assumed to reproduce only asexually. Mating in ascomycetes depends upon functioning mating-type genes that determine compatibility between strains or within a strain. Although homothallic mating, a kind of self-fertility in which mating can occur within one strain, is known in Eupenicillium strains, heterothallic mating, a kind of self-infertility that allows mating only between two strains with different mating types, has not yet been demonstrated in any *Pencillium* species in the subgenus *Pencillium*, but evidence suggests that it does occur in *P. chrysogenum* [8]. While lack of sexual reproduction in *P. dipodomyis* might increase the time to fixation of selectively advantageous alleles, it would simplify the selective effects of Kangaroo Rats and drive local adaptation by maintaining an advantageous combination of alleles, reducing overall genetic variability, and preventing introgression of toxic or immunogenic genes from outside the cheek pouch to seed store transmission environment.

Molecular genetic studies of putatively asexual fungi have consistently detected cryptic sexual cycles and frequently revealed locally adapted populations or cryptic species [9]. Although *P. dipodomyis* has been included in several major studies of species identification and phylogeny in *Penicillium*, it has never been the focus of phylogenetic study, population genetic study, or an evaluation of sexuality [10], [11], [12]. Here, we use phylogenetic and population genetic analysis of 7 molecular markers including mating-type specific markers to examine the patterns of genetic diversity, recombination, and divergence in *P. dipodomyis*. The markers were chosen from four of the five largest supercontigs of the *P. chrysogenum* genome to most likely sample independent portions of the genome. We also conducted a simple *in vitro* evaluation of competitive inhibition between *P. dipodomyis* and other closely related *Penicillium* species detected in cheek pouches.

## Results

**Figure 1:**
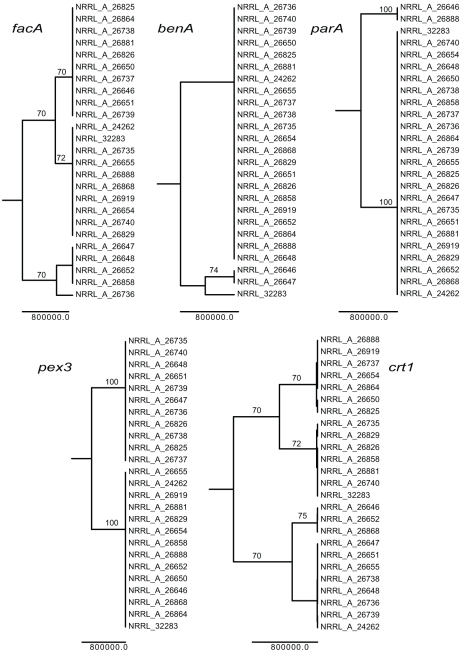
Genealogies of 5 loci from *P. dipodomyis*. Each genealogy shown is the maximum clade credibility tree from BEAST analysis showing nodes with posterior probabilities greater than 0.95, and parsimony bootstrap support values greater than 70% are shown above the nodes.

**Figure 2:**
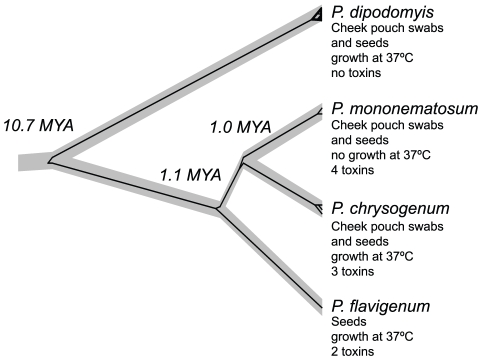
Species tree of *Penicillium* series *Chrysogena* isolates associated with *Dipodomys*. The species tree represents the population history that accounts for diversity within the species and the divergence between them. The grey species tree is supported with posterior probabilities greater than 0.99 at every node and shown containing the *crt1* genealogy inferred along with the species tree in the *BEAST analysis. Median divergence dates are shown above nodes, and characteristics of the species are shown beneath the names.

Sequence data revealed nucleotide diversity (Pi) of 0.16% for all five protein-coding loci and for individual loci diversities of 0.06% (benA), 0.24% (crt1), 0.21% (facA), 0.04% (parA), 0.24% (pex3) were found within 25 isolates of *P. dipodomyis*. Phylogenetic analysis of individual loci revealed conflicting genealogical histories (Fig. 1). Multi-locus species level analysis suggested a divergence of 10.9 MYA (95% HPD 5.6 – 16.4 MYA) for *P. dipodomyis* from other members of *Penicillium* series *Chrysogena* found in association with Kangaroo Rats (Fig. 2). *BEAST analysis of benA alone including sequence data from the industrial fungus *P. nalgiovense* showed that divergence between *P. dipodomyis* and *P. nalgiovense* was less than 1 MYA (95% HPD 0.2 – 2.3 MYA). Assignation of alleles to unique identifiers shows the presence of 20 unique sequence types (ST) of *P. dipodomyis*. Each isolate was either MAT1-1 or MAT1-2, but of the 4 STs represented by more than one isolate, 3 contained both mating-types (Table 1). Both mating-types were found from seed samples and pouch swabs, and overall ratios of MAT1-1 and MAT1-2 in the sample did not differ significantly from a 1∶1 ratio based on a 2×2 contingency table. Overall no significant linkage was detected with a standardized index of association (I_A_) of 0.03 for the five loci excluding MAT and 0.01 including MAT. Pairwise values of I_A_ suggested little linkage between all loci, and although no locus had detectable intra-locus recombination, nearly all pairs of loci failed four-gamete-tests (Table 2), suggesting abundant recombination. In a highly artificial *in vitro* system, growth of *P. dipodomyis* isolates was inhibited by other species (a 44% reduction compared to growth alone) significantly more (p = 0001) than *P. dipodomyis* isolates reduced the growth of other species (25% reduction).

**Table 1:** Mating types and sequence types of *P. dipodomyis* isolates.

ID	Substrate	Mating-type	benA	crt1	facA	parA	pex3	ST
NRRL_32283	Arizona, cheek pouch	1	1	1	1	1	1	1
NRRL_A_26735	Arizona, seed in burrow	**2**	2	1	1	1	2	2
NRRL_A_26740	Arizona, seed in burrow	1	2	1	1	1	2	2
NRRL_A_26829	Arizona, cheek pouch	**2**	2	1	1	1	1	3
NRRL_A_26826	Arizona, cheek pouch	1	2	1	2	1	2	4
NRRL_A_26881	Arizona, cheek pouch	1	2	1	2	1	1	5
NRRL_A_26858	Arizona, cheek pouch	**2**	2	1	3	1	1	6
NRRL_A_26654	Arizona, seed in burrow	**2**	2	2	1	1	1	7
NRRL_A_26888	Arizona, cheek pouch	**2**	2	2	1	2	1	8
NRRL_A_26919	Arizona, cheek pouch	1	2	2	1	1	1	9
NRRL_A_26650	Arizona, seed in burrow	**2**	2	2	2	1	1	10
NRRL_A_26737	Arizona, seed in burrow	**2**	2	2	2	1	2	11
NRRL_A_26825	Arizona, cheek pouch	1	2	2	2	1	2	11
NRRL_A_26864	Arizona, cheek pouch	**2**	2	2	2	1	1	12
NRRL_A_26868	Arizona, cheek pouch	1	2	3	1	1	1	13
NRRL_A_26652	Arizona, seed in burrow	1	2	3	3	1	1	14
NRRL_A_24262	Arizona, seed in soil	1	2	4	1	1	1	15
NRRL_A_26655	Arizona, seed in burrow	1	2	4	1	1	1	15
NRRL_A_26651	Arizona, seed in burrow	1	2	4	2	1	2	16
NRRL_A_26738	Arizona, seed in burrow	**2**	2	4	2	1	2	16
NRRL_A_26739	Arizona, seed in burrow	1	2	4	2	1	2	16
NRRL_A_26648	Arizona, seed in burrow	1	2	4	3	1	2	17
NRRL_A_26736	Arizona, seed in burrow	**2**	2	4	4	1	2	18
NRRL_A_26646	Arizona, seed in burrow	1	6	3	2	2	1	19
NRRL_A_26647	Arizona, seed in burrow	1	6	4	3	1	2	20

**Table 2:** Pairwise I_A_, inter-locus four-gamete-tests, and R_M_ for each locus.

Locus	benA	crt1	facA	parA	pex3	MAT
**benA**	0^A^	0.03^B^	0.01	0.30	−0.02	−0.06
**crt1**	Fail^C^	0	−0.04	0.07	0.06	0.04
**facA**	Fail	Fail	0	−0.06	**0.12** D	−0.04
**parA**	Fail	Fail	Fail	0	−0.03	0.00
**pex3**	Fail	Fail	Fail	**Pass**	0	−0.04
**MAT**	**Pass**	Fail	Fail	Fail	Fail	NA

A Along the diagonal are RM for each locus.

B Above the diagonal are pairwise I_A_ values.

C Below the diagonal are results of the four-gamete-test.

D Significant at *p* = 0.04.

## Discussion

Out results show that *P. dipodomyis* has population structure consistent with sexual recombination despite its supposed asexuality. Although the closely related species *P. chrysogenum* has been shown to have similar levels of diversity on a small spatial scale, *P. chrysogenum* is thought to have globally circulating populations and shows a more clonal population structure [13]. Given the small spatial scale explored here it is quite possible that additional diversity within *P. dipodomyis* is yet to be discovered. Undiscovered genetic diversity might make *P. dipodomyis* more diverse than closely related *Penicillium* species with global populations. Our finding suggests that recombination is frequent in *P. dipodomyis*. This population structure allows for more effective natural selection, but also forces the disruption of favorable genetic combinations via recombination. The timing of recombination and any genotypic amplification of the asexual stage will influence which ecological factors play a role in determining how much selection acts independently on sexual and asexual spores.

The lack of demonstration of any heterothallism in the subgenus *Penicillium* is likely to be a result of the difficulty in observing microscopic morphological events despite their frequency in natural environments. Few observations of natural microscopic communities have been made on seeds, but this is possible with scanning electron microscopy [14]. It would be similarly possible to sample fur from the lined pouches of Kangaroo Rats for electron microscopy. Finding the conditions that result in successful recapitulation of a naturally occurring sexual cycle remains elusive across Eurotiomycete fungi, but our results add to the mounting evidence that the large number of species in *Penicillium* and *Aspergillus* that are putatively asexual are much more likely to be cryptically promiscuous [12], [15], [16], [17], [18], [19], [20], [21]. More observations of natural populations may reveal sexual stages of commonly isolated but rarely observed *in situ* fungi.

*P. dipodomyis* is one of a many *Penicillium* species associated with *Dipodomys* cheek pouches and seed storage, but it is the only one capable of growth at 37°C and producing no known toxins. Our analyses that suggest that the *P. dipodomyis* lineage diverged 10.7 MYA from other *Penicillium* series *Chrysogena* species also found in association with *Dipodomys* are intriguing given the estimated divergence of *Dipodomys* from its sister taxa 11.4 MYA [22]. The much more recent divergence of *P. dipodomyis* from *P. nalgiovense* does not support a long association between the *P. dipodomyis* lineage and Kangaroo Rats, but the ecology and origin of *P. nalgiovense* is unclear, and it is known exclusively from meat and cheese preparation. Several putative isolates of *P. nalgiovense* from sandy desert soil in California, USA, were revealed to be more closely related to *P. dipodomyis* with DNA sequence data. The inability of *P. nalgiovense* to grow at 37°C suggests that although this species may be able to colonize some rodent foodstuffs without producing known toxins, it is unlikely to be actively growing in small mammal bodies. However, we cannot rule out that *P. nalgiovense* evolved from a *Penicillium* species similar to *P. dipodomyis* already associated with desert rodents.

Desert environments are precarious for small mammals because dry seeds are thought to make up nearly all of Kangaroo Rat diets, and the relationship between water availability fungal colonization of seeds and toxin production may seriously effect *Dipodomys* survival. Humans use the *P. nalgiovense* primarily to protect aged meat and cheese from colonization by other toxic fungi, and Kangaroo rats might benefit from similar protective effects of *P. dipodomyis*. However, production of potentially toxic secondary compounds may give *Penicillium* species a competitive advantage, and indeed in our highly artificial but secondary compound promoting *in vitro* assays, *P. dipodomyis* was a relatively weaker competitor compared to the other *Penicillium* species found in cheek pouches. In natural competitive environments of cheek pouches and low water potential seeds, *P. dipodomyis* might be a stronger competitor, and many competitive advantages of the other species could also be overcome by higher abundance or prior occupancy of *P. dipodomyis* in the initial colonization environment. Abundant growth at mammalian body temperature despite lacking production of toxic chemicals could give *P. dipodomyis* an advantage in exploiting Kangaroo Rat cheek pouch environments while allowing Kangaroo Rats to benefit from seed colonization by nontoxic fungi.

Resource competition can lead to speciation particularly when linked to mating success [23]. If mating frequency is contact rate dependent then cheek pouches and stored seeds may function as a population concentrator for *Penicillium* strains that would be unlikely to encounter each other on more diffuse substrates. In the case of *P. dipodomyis*, mating success is currently an unknown quantity, but the mammal hosts should not be discounted as a recombination arena since both mating-types are found in cheek pouch swabs. One potential danger to kangaroo rats would be the mating success of strains that produce toxins or other undesirable traits. This effect could be negated if recombination is restricted to occur in environments where mating strains must coexist with healthy Kangaroo rats. Furthermore, adaptation of fungi to mammalian host niches is of growing interest as evidence mounts that fungal diseases evolve to respond to host environments and immune responses even when they also have non-mammalian environmental reservoirs [24].

## Materials and Methods

### Sampling, Primers and PCR

31 isolates from rodent burrows or cheek pouches were obtained from the NRRL from across a small spatial scale near Portal Arizona. Isolates were grown on Malt Extract Agar (Oxoid) for 7 days and its identity checked based on morphology and BLAST of beta-tublin sequences. 1cm cubes of agar from the growing edge of the fungal colony was transferred into sterile distilled water for storage while a loop of conidia was used to grow each isolate in 1.5 ml of Malt Extract Broth (Oxoid) for 4 days at 30°C, and approximately 30 mg of hyphae were placed into tubes with silica beads for DNA extraction using DNeasy Plant Mini Kits (Qiagen). Based on BLAST of beta-tubulin sequences of the 31 isolates several species were detected including three isolates of *P. chrysogenum*, 2 isolates of *P. mononematosum*, and one isolate of *P. flavigenum.* These isolates were included in species level phylogenetic analysis to determine the relationship between these co-occurring species. Mating-type primers were designed based on *P. chrysogenum* gene sequences in GenBank and published *Penicillium* mating-type sequences [12] to result in amplification of MAT1-1 or MAT1-2 amplicons specific to a forward primer embedded in conserved portions of the MAT locus, PdAspMat1f (5′-CGACTGGATGTGTTGGGCA-3′), PdAspMat2f (5′- CCACCATTTGGTCAAGG-3′), PdAspMatR (5′-AAAGTCGGCAGAAAGATTCA-3′). Other primers were designed based on *P. chrysogenum* sequences for five protein coding genes calreticulin (*crt1*) [crt1f (5′-AAGAAGATYGAYAACAAGGGCAAGAC-3′), crt1r (5′-TGGAAGTGGTAYTTYGAG-3′)], acetyl-CoA (*facA*) [facAf (5′TCRGGCTTCTCGGCCTC3′), facAr (5′-ACACGRCCRCGGATCCAGTA-3′)], peroxin-3 (*pex3*)[pex3f (5′-CGCTGGTTNCRRCGCAATCGCA-3′), pex3r (5′-TGGTTYTGTTCGAANCGTCG-3′)], beta-tubulin (*benA*) [benAf (5′-GTAACCAAATCGGTGCTGCTTTC-3′), benAr (5′-CCCTCAGTGTAGTGACCCTTGGC-3′)], and phosphoadenosine phosphosulphate reductase (*parA*) [parA (5′-CCCGAGATTGTNTTCACCAA3′), and parAr (5′-ACCTTGGCNACCCAGTCGTA-3′)]. We used *TopTaq* polymerase and reagents (Qiagen) with a touchdown PCR protocol. After an initial temperature of 95°C for 30 seconds, the 30-second long annealing temperatures were lowered 1 degree each cycle for 15 cycles from 65°C to 50°C followed by 33 cycles at 50°C. For product extension we used a hold at 72°C for 2 minutes before finally returning to 95° for another cycle. We used standard sequencing protocols with the same primers used in PCR. Sequences were deposited in GenBank (Accession nos. JF714257-JF714411).

### Phylogenetic and population genetic analysis

Sequence data were aligned manually in MacClade 4.05 [25] and each locus was analyzed separately to detect concordance and discordance among genelaogies, a hallmark of recombination [26], [27], [28]. We used PAUP* 4.10b [29] for parsimony analysis and BEAST [30]for Bayesian analysis of each locus. Because of the small number of genotypes, we were able to use branch and bound searches for parsimony analysis and excluded invariant sites. For BEAST analysis, we used HKY substitution models with invariant sites, a fixed molecular clock, a constant population size coalescent tree prior, a UPGMA starting tree and a chain of 10 million generations logging parameters every 1000 generations. Nodes with greater than 70% bootstrap support or 95% posterior probability were considered statistically supported. To infer relationships between closely related species found in association with *Dipodomys* and divergence from *P. dipodomyis* we used the *BEAST approach of simultaneous estimation of genealogy and phylogeny [31]. *BEAST uses coalescent expectations of genealogical histories to allow conflicting topologies between genealogies and between genealogies and species histories. We used all five protein coding genes with unlinked HKY plus invariant substitution models, a Yule process species tree model, and a piecewise linear with constant root population size for the underlying coalescent model for the genealogies. We sampled from a chain of 100 million generations saving values every 10000 generations to estimate parameters. For the benA analysis we used the same parameters sampling from 10 million generations and saving values every 1000 generations. We inferred divergence dates based on a molecular clock assumption of 3.0×10^9^ substitutions per year, a rate estimated for Eurotiomycete fungi [32]. We used the program dnaSP v5 [33] to estimate nucleotide diversity and the minimum number of recombination events (R_M_), and we used the program LIAN [34] to assess clonality by estimating standardized index of association I_A_.

### Competitive experiments

**Figure 3:**
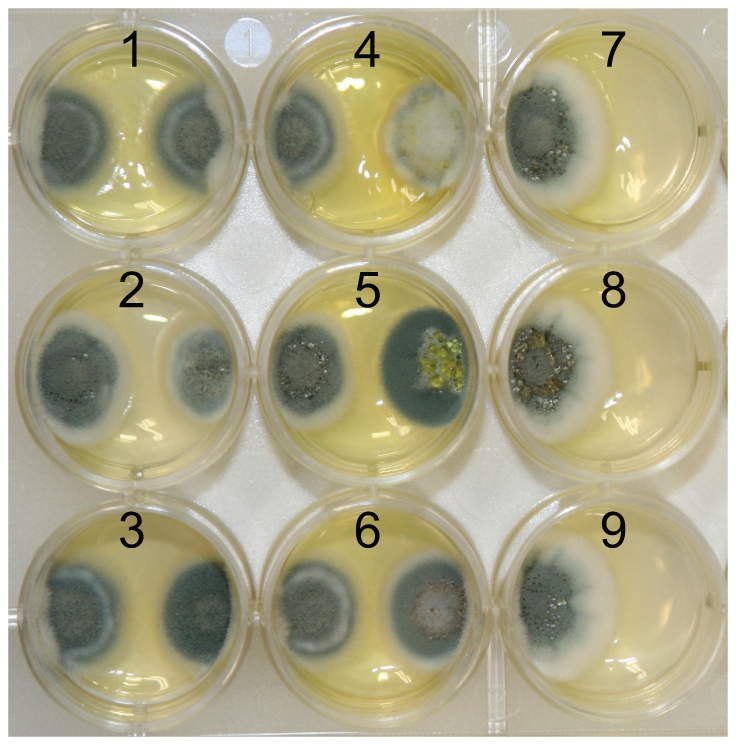
Competitive effects on *P. dipodomyis* growth *in vitro*. This example plate of *P. dipodomyis* strain NRRL 26881 shows how growth assays were made. Along the left side of the plates are repeated inoculations of NRRL 26881, while along the right side of the plates are other strains. The numbers 1–9 correspond to different inoculations. Numbers 1–3 include the strains of *P. dipodomyis*, NRRL 26881, 26864, 26829, respectively, while 4–6 include the other species, *P. chrysogenum*, *P. flavigenum*, and *P. mononematosum*, respectively. Numbers 7–9 include no other strains and are used to calculate the effect.

To measure competitive effects we inoculated each side of 30 mm agar dish containing Czapek-Dox agar (Oxoid) with a 0.5 µl suspension of 1×10^7^ conidia/ml. We used three isolates of *P. dipodomyis* and one isolate each of *P. chrysogenum*, *P. flavigenum* and *P. mononematosum*. Isolates were then grown at 27°C for 7 Days. The total growth area of each isolate was measured using Image J, and the competitive effect of an isolate was measured as the difference between growth area of the inhibited isolate alone minus growth in the presence of the isolate divided by growth alone (Figure 3). This is an appropriate measure of fitness because the surface area of the colony is directly related to the number of spores formed by an isolate. Each pair of isolates was tested in triplicate. T-tests were used to compare the effect of *P. dipodomyis* on other *Penicillium* species and *vise versa*.
